# Hypercalcemia associated with isolated bone marrow sarcoidosis in a patient with underlying monoclonal gammopathy of undetermined significance: case report and review of literature

**DOI:** 10.1186/s40364-016-0072-5

**Published:** 2016-09-15

**Authors:** John Gubatan, Xiaohui Wang, Abner Louissaint, Anuj Mahindra, John Vanderpool

**Affiliations:** 1 Department of Medicine, Beth Israel Deaconess Medical Center and Harvard Medical School, 330 Brookline Avenue, Boston, MA 02215 USA; 2 Hallmark Health Medical Associates, Melrose-Wakefield Hospital, Reading, MA USA; 3 Department of Pathology, Massachusetts General Hospital, Boston, MA USA; 4 Division of Hematology and Oncology, University of California San Francisco Medical Center, San Francisco, CA USA; 5 Department of Medicine, Massachusetts General Hospital and Harvard Medical School, Boston, MA USA

**Keywords:** Hypercalcemia, Bone marrow sarcoidosis, Monoclonal gammopathy of underdetermined significance, Lymphoproliferative disorders

## Abstract

**Background:**

Bone marrow sarcoidosis is extremely rare. The association between sarcoidosis and lymphoproliferative disorders has been previously speculated, although the diagnosis of sarcoidosis often precedes any hematological derangements.

**Case presentation:**

Here, we report for the first time, a case of a 57-year-old Caucasian woman with a previous diagnosis of monoclonal gammopathy of undetermined significance (MGUS) developing hypercalcemia and renal failure with workup notable for isolated bone marrow sarcoidosis and not multiple myeloma as expected. The patient was successfully managed with prednisone taper therapy with resolution of her hypercalcemia and repeat bone marrow biopsies demonstrating resolving granulomas.

**Conclusions:**

Our case illustrates the diagnostic challenges associated with bone marrow sarcoidosis and suggest that chronic immune stimulation in the bone marrow in the setting of MGUS may be associated with the development of localized sarcoidosis. The long term consequences of steroid therapy targeting sarcoidosis in this patient with underlying MGUS remain unknown. Greater surveillance and closer followup is planned in light of the increased risk of malignant transformation of MGUS into multiple myeloma in the setting of bone marrow sarcoidosis.

## Background

Extrapulmonary manifestations of sarcoidosis are uncommon. Bone marrow sarcoidosis is infrequently clinically encountered. In the few cases reported, bone marrow involvement most often occurs in the setting of systemic sarcoidosis [1–3]. Isolated bone marrow sarcoidosis is exceedingly rare [4, 5]. The association between sarcoidosis and malignant disease including lymphoproliferative disorders has been previously explored [6, 7]. It has been postulated that chronic immune stimulation associated with chronic active sarcoidosis may lead to lymphoproliferative disorders, termed the “sarcoid-lymphoma syndrome” [7, 8]. In contrast to this temporal relationship, patients with hematologic malignancies for more than a year have been observed to develop sarcoidosis [9]. Herein, we report an unusual case of a patient with four-year history of monoclonal gammopathy of undetermined significance (MGUS) preceding the diagnosis of isolated bone marrow sarcoidosis presenting as hypercalcemia.

## Case presentation

A 57-year-old Caucasian woman with a four-year history of monoclonal gammopathy of undetermined significance (MGUS) complained of several weeks of dry mouth, polydipsia and episodes of confusion. Her serum calcium was 14.4 mg/dL when tested at the office of her primary care physician (PCP) and sent to the emergency department (ED) where her exam was remarkable only for dry mucous membranes. Initial labs were notable for Ca 14.4 mg/dL, albumin 5.0 g/dL, PTH 7 pg/mL, and Cr at 1.94. CBC was notable for mild pancytopenia with WBC 3.5 (th/cmm) with normal differential, Hgb 11.7 g/dL, Hct 34.2 (%), and PLT 145,000 (th/cmm). She received a bolus of normal saline and was subsequently admitted to inpatient service. Initially, the patient’s hypercalcemia was attributed to milk-alkali syndrome and her calcium supplements were discontinued. The patient was managed only with IV hydration. The patient’s serum calcium trended down to 10 mg/dL at the time of discharge.

**Fig. 1:**
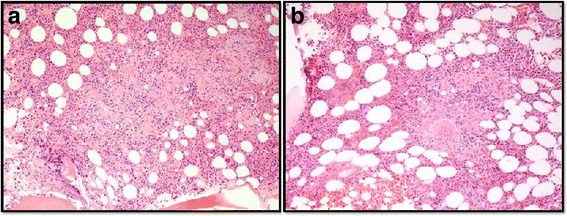
Bone marrow biopsies demonstrating non-necrotizing granulomas before steroid therapy (100× original magnification) (**a**) and follow-up bone marrow biopsy 5 months after diagnosis (4 months later after steroid therapy) shows partial resolution of granulomas without evidence of malignancy (200× original magnification) (**b**)

**Fig. 2:**
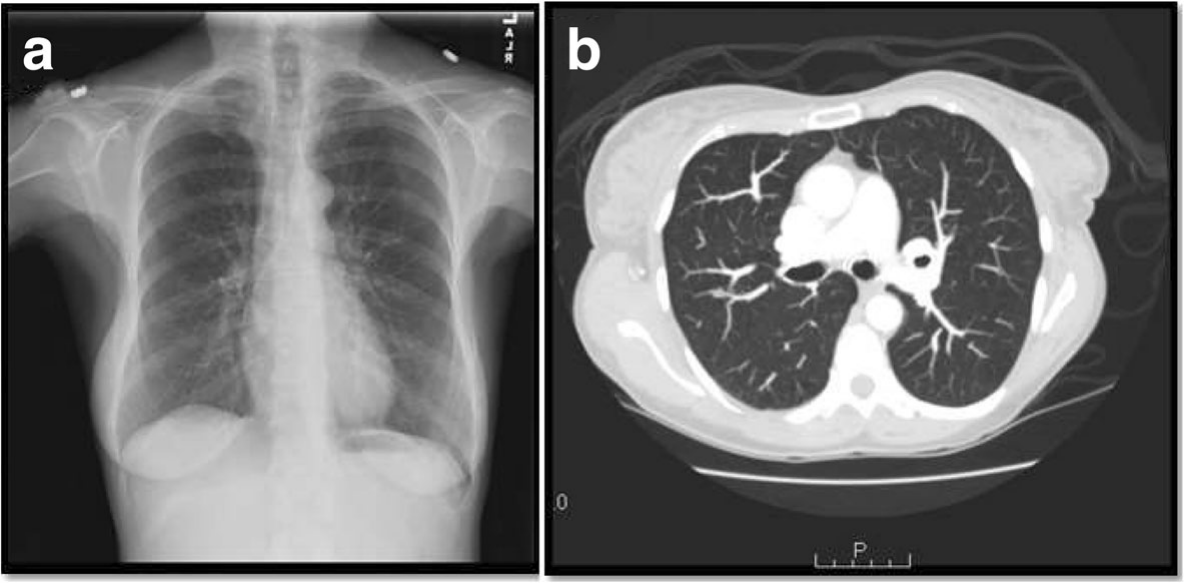
Imaging Results: Normal Chest X-Ray (**a**) and Chest CT without evidence of intrathoracic lymphadenopathy or pulmonary parenchymal lesions (**b**)

There was a concern for a neoplastic process, particularly myeloma, given her history of MGUS. The patient underwent bone marrow biopsy at the time of discharge, which was remarkable for non-necrotizing granulomas with no findings suggestive of plasma cell dyscrasia or other neoplasms. (Fig. 1a) Two weeks later, the patient’s repeat calcium was 12.7 mg/dL despite adequate fluid intake. The patient was admitted for further evaluation of hypercalcemia and bone marrow granulomas. An extensive hypercalcemia workup was unrevealing. Malignant and infectious etiologies of granulomatous disease in this patient were investigated. Results from repeat SPEP, UPEP, and serum free light chains were unchanged from baseline values. PTHrp was undetectable. PET scan showed no FDG avid lesions. Infectious workup for granulomas was negative. The patient’s hypercalcemia was attributed to the granulomas given exclusion of other potential causes. As a result, a workup for sarcoidosis was pursued, which was notable for elevated ACE 126 and urinary calcium of 256 mg/24 h (Table 1). Chest X-ray and chest CT revealed no intrathoracic lymphadenopathy or pulmonary parenchymal findings (Fig. 2). There was no evidence of sarcoidosis in any other organ.

**Table 1:** Calcium studies during patient’s two hospitalizations

Lab Value (Units)	1st Admission	2nd Admission	Normal Values
Ca (mg/dL)	14.3	12.7	8.5–10.5
iCa (mg/dL)	1.73		1.14–1.30
PTH (pg/mL)	7	5	10.0–60.0
Urine Ca	–	256 mg/24 h	Female: < 250 mg/24 h
1,25 OH Vit D (ng/mL)	–	49 pg/mL	–
25 OH Vit D (ng/mL)	44 ng/mL	–	25–80
PO4 (mg/dL)	5	2.5	2.6–4.5
Albumin (g/dL)	5	5	3.3–5.0
Creatinine	1.94	1.23	0.60–1.50

The patient underwent a rapid prednisone taper over 9 days. After discontinuation of prednisone, she developed hypercalcemia again. She was then treated with a slow prednisone taper for four months with normalization of serum calcium levels. A follow-up bone marrow biopsy 5 months later (1 month after 4-month course of slow prednisone taper) showed partial resolution of granulomas without malignancies. (Fig. 1b) At her follow-up PCP visit, two years after her initial presentation, the patient was overall asymptomatic with normal serum calcium, ACE, and CBC.

## Discussion

Bone marrow sarcoidosis is rare and previously reported cases mostly involved patients with severe, chronic, and widespread sarcoidosis. Bone marrow biopsies are not routinely performed in the standard workup of sarcoidosis, thus the overall incidence of bone marrow involvement is unknown. One small cohort study [5] estimated the bone marrow involvement of patients with sarcoidosis to be 10 % with most patients exhibiting hematologic abnormalities as well as systemic sarcoidosis.

Our case illustrates the diagnostic challenges associated with an atypical and rare manifestation of sarcoidosis. The clinical presentation of bone marrow sarcoidosis is variable, non-specific, and may overlap with those of systemic sarcoidosis which include fever, fatigue, malaise, weight loss, and night sweats. Patients with bone marrow involvement may have associated hematological abnormalities including anemia, leukopenia, and thrombocytopenia [3–5].

Although the diagnosis of sarcoidosis seemed unlikely during the early workup of this patient, three lab abnormalities had some clinical utility and diagnostic value in support of sarcoidosis: elevated ACE serum level, hypercalcemia, and hypercalciuria.. ACE levels are elevated in 75% of untreated patients with sarcoidosis and levels tend to correlate with disease activity [10]. One study by Ackermann et al. [11] estimates the prevalence of hypercalcemia in patients with sarcoidosis to be around 2 to 63 % depending on the population studied. The study attributed the differences in prevalence of hypercalcemia among sarcoidosis patients to the undulating course of subacute sarcoidosis as well as variability in testing for serum calcium. The study also found that hypercalciuria appeared to be twice as prevalent as hypercalcemia and argued that checking urine calcium should be part of the workup of sarcoidosis.

Given the rare occurrence of bone marrow sarcoidosis, there are currently no randomized controlled trials comparing treatment modalities. However, a review of the relevant literature presents four case reports of patients with biopsy-proven bone marrow sarcoidosis demonstrating various approaches to treatment. One case report by Saliba et al. [12] involved a 71-year-old. woman with severe hypercalcemia and isolated BM sarcoidosis. The patient was treated with IV saline hydration and her hypercalcemia resolved after five days. The patient did not undergo steroid therapy as her hypercalcemia did not reoccur. Another case report by Slart et al. [13] involved a 49-year-old man with lytic lesions and symptomatic hypercalcemia found to have bone marrow sarcoidosis and subacute pulmonary sarcoidosis. The patient responded well to a steroid taper. A third case report by del Mar Osma et al. [14] involved 44-year-old woman with isolated bone marrow sarcoidosis presenting with weakness, weight loss, and nightly fevers. The patient was started on adalimumab with subsequent resolution of her constitutional symptoms. The final case report by Patel et al. [15] involved a 42-year-old man with diabetes and systemic sarcoidosis with bone marrow involvement presenting with anemia, thrombocytopenia, and splenomegaly. The patient was initially treated with steroids, but discontinued due to complications with his diabetes. The patient was treated with adalimumab and his hematologic parameters improved after two courses of therapy.

These case reports highlight that the therapeutic approach to patients with bone marrow sarcoidosis depend on clinical presentation. Patients with symptomatic hypercalcemia appear to respond well to IV saline hydration and in severe cases may require steroid taper therapy. Ackermann et al. [11] recommended starting corticosteroid treatment if total corrected calcium rises beyond 3 mmol/l (12 mg/dl) or at lower levels if patients become symptomatic. Patients with constitutional symptoms, systemic sarcoidosis, or contraindications to steroid therapy may respond better to management with adalimumab. In our patient, IV saline therapy had minimal effects on serum calcium. The patient was started on a rapid steroid taper for 10 days with improvements in serum calcium. Upon discontinuation of the steroids, the patient’s hypercalcemia reoccurred. The patient was managed with a second, but slow taper of steroids for four months. The patient’s hypercalcemia and serum ACE levels normalized and continue to remain stable.

An intriguing aspect of this case report involves the potential relationship with the patient’s underlying MGUS. The association between sarcoidosis and the increased risk of malignancy particularly lymphoproliferative disorders has been previously examined [6–8] and case reports with patients with sarcoidosis and MGUS and multiple myeloma have been previously reported. Likewise, it has been hypothesized that the risk of multiple myeloma is increased in patients with sarcoidosis and a more rapid progression from MGUS to multiple myeloma has been observed in the few cases reported [14–16]. In virtually all of these cases, the diagnosis of sarcoidosis preceded or occurred concurrently with that of MGUS. In our patient, the diagnosis of sarcoidosis occurred four years after that of MGUS. It may be possible that the patient developed subacute bone marrow sarcoidosis years before her diagnosis of MGUS, but only came to attention recently after presenting with hypercalcemia. Alternatively, both conditions may have developed concurrently, with her bone marrow sarcoidosis remaining quiescent until her hypercalcemia became clinically significant. Finally, our patient’s case may represent a situation in which MGUS preceded the development of sarcoidosis. Indeed, a review of her CBC through the years including those years prior to her admission when she was only diagnosed with MGUS would seem to reinforce this later point. (Table 2) Previous CBCs were normal for multiple preceding years and she only had pancytopenia on presentation for admission with hypercalcemia secondary to bone marrow isolated sarcoidosis.

**Table 2:** Complete blood count (CBC) studies in preceding years, upon admission, and post-steroid treatments

Hematological Parameters (units)	4 years Before Admission 7/22/2006	3 years Before Admission 10/15/2007	Last CBC Before Admission 5/6/2008	At Admission 8/25/2010	At Discharge 9/3/2010	After 1st steroid taper 09/20/10	After 2nd steroid taper 01/18/11	PCP Visit 6/13/2012	Normal Values
HGB (g/dL)	13.7	14.1	12.3	11.7	11.5	10.4	12.8	13.5	12.0–16.0
HCT (%)	39.4	42	35.8	34.2	33.5	31	37.5	40.6	36.0–46.0
PLT (th/cmm)	198	225	193	145	210	198	157	188	150–400
WBC (th/cmm)	9.2	6	6.4	3.5	3.6	7.4	4.7	3.8	4.5–11.0
Poly (%)	79	60	–	61	62	94	83	57	40–70
Lymphs (%)	18	34	–	28	25	5	13	33	22–44
Monos (%)	3	4	–	10	11	1	3	8	4.0–11.0
Eos (%)	0	1	–	1	1	0	0	1	0–8
Basos (%)	0	1	–	0	1	0	1	1	0–3

Sarcoidosis has been observed to develop in patients with hematologic malignancies with some investigators attributing this to the use of immunosuppressive therapy targeted at the underlying malignancy [17, 18]. Aside from her known MGUS, our patient has never had any malignant disease and was never treated with chemotherapy or immunosuppressives. This raises the possibility that MGUS, in addition to hematologic malignancies, may also be a risk factor in the development of sarcoidosis. It may be possible that the abnormal bone marrow milieu in this patient with MGUS had an impact on the local development of granulomas.

## Conclusions

In conclusion, isolated bone marrow sarcoidosis is rare and can be a diagnostic challenge. The clinical suspicion for sarcoidosis should be raised in patients with underlying hematologic disorders or malignancies. Due to the rarity of the condition, long terms outcomes in such patients are unclear. Surveillance and regular follow-up is planned in view of the possibility of progression to multiple myeloma or another lymphoproliferative disorder.

## Abbreviations

ACE: Angiotensin converting enzyme
Ca: Calcium
CBC: Complete blood cell
Cr: Creatinine
CT: Computed tomography
ED: Emergency department
FDG: Fluorine-18-fluorodeoxyglucose
Hct: Hematocrit
Hgb: Hemoglobin
MGUS: Monoclonal gammopathy of undetermined significance
PCP: Primary care physician
PET: Positron emission tomography
Plt: Platelets
PTH: Parathyroid hormone
PTHrp: Parathyroid hormone related protein
SPEP: Serum protein electrophoresis
UPEP: Urine protein electrophoresis
WBC: White blood cell
